# Self-limiting isolated choroidal granuloma with serous retinal detachment: atypical cat scratch disease without feline exposure

**DOI:** 10.1186/s12886-026-04711-1

**Published:** 2026-03-25

**Authors:** Yuting Peng, Lu Wang, Bingqian Zhou, Waner Lin, Long Pang

**Affiliations:** Department of Ophthalmology, The Second Affiliated Hospital of Guangzhou University of Chinese Medicine, Guangdong Provincial Hospital of Chinese Medicine, Guangzhou, Guangdong 510120 China

**Keywords:** Choroidal granuloma, Serous retinal detachment, Ocular bartonellosis, Cat scratch disease, Multimodal imaging

## Abstract

**Background:**

The ocular manifestations of cat scratch disease (CSD) are diverse. We report a rare case of isolated choroidal granuloma with serous retinal detachment (SRD) secondary to *Bartonella henselae* infection in a patient without feline exposure.

**Case presentation:**

A retrospective analysis of a 38-year-old female presenting in March 2024 with unilateral visual impairment and metamorphopsia. Diagnostic evaluations included multimodal imaging (spectral-domain optical coherence tomography [SD-OCT], fluorescein angiography [FA], indocyanine green angiography [ICGA]), and serologic testing. Ocular examination revealed a yellowish-white subretinal lesion with associated SRD superotemporal to the optic disc. SD-OCT demonstrated a dome-shaped choroidal elevation with homogeneous hyporeflectivity and subretinal fluid. FA showed late hyperfluorescence of the lesion, while ICGA revealed persistent hypofluorescence. Serologic testing confirmed elevated *Bartonella henselae* IgG titers (1:256). The patient had no systemic symptoms or history of feline contact and initially received systemic steroids, topical anti-inflammatory/antibiotic agents, and traditional Chinese medicine. All treatments were discontinued after 24 h, and the lesion subsequently resolved with complete resolution of subretinal fluid and best-corrected visual acuity (BCVA) recovery to 20/20.

**Conclusion:**

Ocular bartonellosis may manifest as an isolated choroidal granuloma with vision-threatening SRD, even in the absence of feline exposure. Multimodal imaging and serologic testing are critical for diagnosis. The condition may resolve spontaneously without targeted antimicrobial therapy for *Bartonella* infection, even in the absence of anti-inflammatory therapy.

## Background

Cat scratch disease (CSD) is caused by infection with the bacteria *Bartonella henselae*, classically presents with regional lymphadenopathy following feline contact. Atypical CSD accounted for 1.5% of all cases, resulting in an average annual incidence of 0.7 cases/100,000 population [1]. Approximately 5–10% of *Bartonella henselae* infections develop ophthalmologic complications [2, 3]. Ocular manifestations of CSD demonstrate a tripartite pattern of involvement: (1) Inflammatory manifestations: Parinaud oculoglandular syndrome, neuroretinitis, retinochoroiditis, (2) Vasculopathic events: branch retinal artery occlusion, paracentral acute middle maculopathy, retinal vasoproliferative tumors, and (3) Structural complications: exudative maculopathy, pseudometastatic choroiditis, endophthalmitis [4–7]. Its polymorphic ocular presentations pose significant diagnostic challenges, frequently requiring differential diagnosis from infectious etiologies, non-infectious inflammatory conditions, and neoplastic processes. The severity of systemic and ocular manifestations correlates with immunity status of the host and pathogen-related factors [8–10]. Choroidal granulomas with exudative retinal detachment due to *Bartonella henselae* infection have been rarely reported [11, 12].

We present a rare case of self-resolving an isolated choroidal granuloma with associated SRD representing an atypical manifestation of CSD in the absence of feline exposure.

## Case presentation

A 38-year-old woman presented to the Department of Ophthalmology at Guangdong Provincial Hospital of Chinese Medicine with a one-day history of a dark shadow obscuring vision in her right eye. Initial ophthalmic evaluation revealed best-corrected visual acuity (BCVA) of 20/20 in both eyes. Detailed inquiry confirmed no history of pet exposure (e.g., cats, dogs, scratches or bites), travel to endemic areas, or relevant special contact. Fundoscopic examination demonstrated a normal left eye, while the right eye exhibited a disc-sized yellowish-white subretinal lesion. Anterior segment examinations were unremarkable bilaterally, with no evidence of intraocular inflammation, vitreous cells, retinal vasculitis, or retinitis.

Within 48 h of presentation, the patient experienced progressive visual deterioration in her right eye, with BCVA decreasing to 20/25 in right eye, while maintaining 20/20 in the left eye. The scotoma expanded concomitantly with the visual decline, demonstrating rapid lesion progression.

The differential diagnosis for an isolated choroidal mass encompasses a wide spectrum of etiologies including inflammatory conditions, neoplastic processes, and infectious diseases. To establish a definitive diagnosis, we implemented a comprehensive diagnostic approach incorporating detailed clinical evaluation, multimodal imaging, and laboratory investigations.

### Ocular multimodal imaging findings

**Fig. 1:**
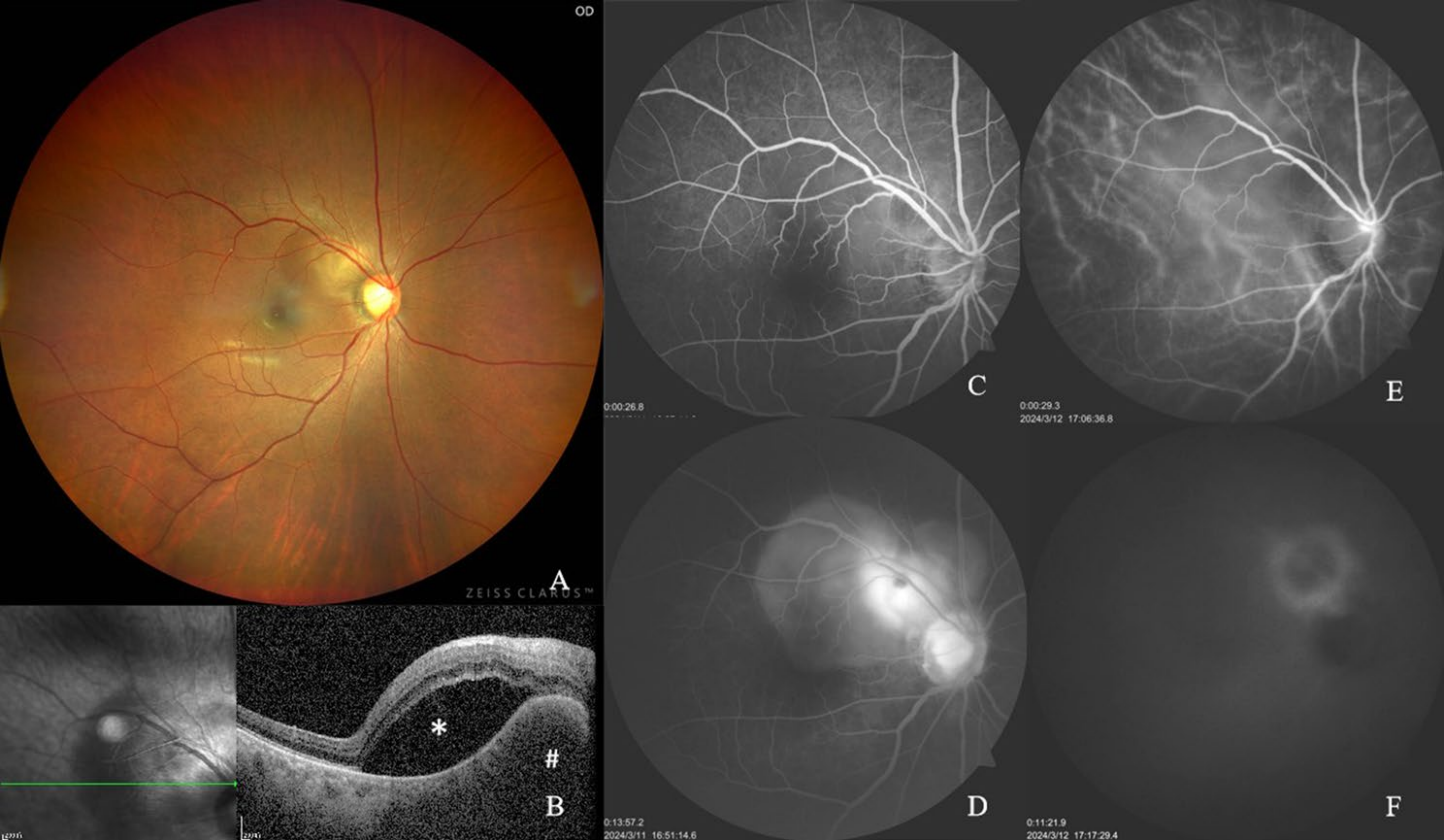
Multimodal Fundus Imaging at Disease Onset. (**A**) Color fundus photography shows a well-circumscribed, yellowish-white lesion (~ 1 disc diameter in size) located superotemporal to the optic disc, associated with overlying localized retinal detachment. (**B**) Spectral-domain OCT demonstrates a dome-shaped choroidal elevation exhibiting homogeneous hyporeflectivity, accompanied by secondary serous retinal detachment (SRD). (**C**-**D**) Fluorescein angiography (FA) sequences show early-phase hypofluorescence of the lesion (**C**), progressing to late-phase hyperfluorescence with neuroepithelial detachment and optic disc staining (**D**). (**E**-**F**) Indocyanine green angiography (ICGA) identifies persistent hypofluorescence across all angiographic phases, demarcated by a late-stage annular hyperfluorescent border (**E**: early-phase; **F**: late-phase). # reprensents Choroidal Granuloma; * reprensents Subretinal Fluid

Fundus photography demonstrated a well-demarcated, yellowish-white lesion measuring approximately one disc diameter, located superotemporally to the optic disc, accompanied by an overlying localized SRD (Fig. 1A).

**Fig. 2:**
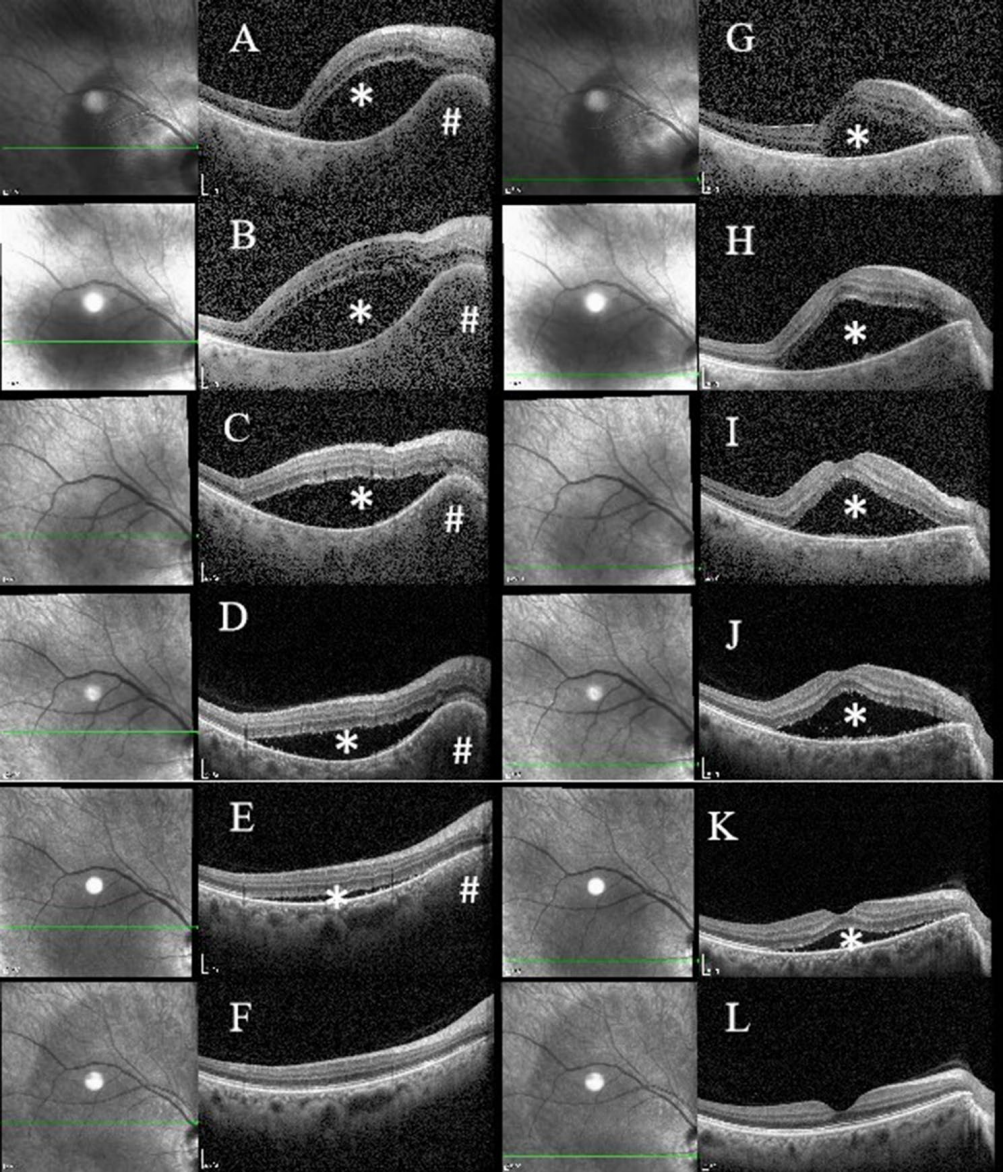
Longitudinal OCT Imaging of Choroidal Granuloma Evolution and Associated Subretinal Fluid Dynamics. **A**-**F** illustrate lesion morphology at the granuloma site at sequential timepoints: 3 days, 1 week, 9 days, 2weeks, 3 weeks, and 11 weeks post-onset. Corresponding macular fovea changes are shown in **G**-**L** at identical intervals. Initial progression is characterized by granuloma enlargement with concomitant subretinal fluid expansion during the first week (**A**, **B**, **G**, **H**). Subsequent resolution phases reveal gradual lesion regression beginning in the second week (**C**, **D**, **I**, **J**), near-complete resolution by the third week (**E**, **K**), and ultimate restoration of normal anatomical architecture at the 11-week follow-up (**F**, **L**). # reprensents Choroidal Granuloma; * reprensents Subretinal Fluid

SD-OCT imaging identified a dome-shaped choroidal elevation with homogeneous hyporeflectivity and accompanied by a secondary SRD (Figs. 1B and 2).

FA showed initial hypofluorescence of the lesion during early phases (Fig. 1C), progressing to late-phase hyperfluorescence with neuroepithelial detachment and optic disc staining (Fig. 1D).

ICGA revealed persistent hypofluorescence throughout all phases, bordered by a distinct annular hyperfluorescent rim in late stage (Fig. 1E and F).

Ocular B-scan ultrasonography revealed a localized retinal detachment in the right eye, as well as a small choroidal lesion with low reflectivity and minimal vascular flow near the superotemporal optic disc.

Chest X-ray were normal.

Chest CT demonstrated benign lesions with no evidence of tuberculosis, and regular follow-up was recommended.

### Laboratory findings

Routine examinations including urinalysis, stool routine, biochemistry and coagulation function were normal. Serum tumor markers (CEA, AFP, CA125, CA15-3, CA19-9) were negative. Autoimmune, rheumatologic and immunological examinations were negative. Syphilis serology was negative. Tuberculosis-related tests including PPD test, tuberculosis interferon-gamma release assay, tuberculosis antibody and tuberculosis complex nucleic acid test were negative. Erythrocyte sedimentation rate (ESR) was mildly elevated at 37 mm/h (reference range: 0–32 mm/h). Peripheral blood eosinophil percentage (8.9%, reference range: 0.4-8.0%) and absolute eosinophil count (0.56 × 10⁹/L, reference range: 0.02–0.52 × 10⁹/L) were slightly increased.

The elevated eosinophil count initially raised suspicion for parasitic etiology, so relevant serological tests were performed. Serum Toxocara antibody and Toxoplasma gondii (TOX) antibody were negative, thus excluding the most common parasitic causes of ocular granulomatous lesions in our clinical setting.

Taken together, the clinical, multimodal imaging, and laboratory findings support the diagnosis of choroidal granuloma and help exclude other diagnostic considerations, including choroidal tumor and tuberculous granuloma.

These findings initially guided consideration of anti-inflammatory therapy.

Initial therapy comprised:

1. Systemic/intravenous dexamethasone sodium phosphate.
2. Topical tobramycin-dexamethasone and diclofenac sodium eye drops.
3. Adjunctive traditional Chinese medicine (Shenling Baizhu Powder: A classic Chinese herbal formula for fortifying spleen Qi and resolving dampness).

Disease progression occurred at 24 h post-treatment, evidenced by increased subretinal fluid (Fig. 2B and H). Immediate cessation of all anti-inflammatory therapies preceded subsequent clinical improvement. Subsequent monitoring revealed: one week later, gradual regression of the choroidal nodule and resolving subretinal fluid (Fig. 2C, I, D and J). About three weeks later: Follow-up imaging demonstrated completely resolved choroidal granuloma and nearly complete resolution of subretinal fluid (Fig. 2E and K), and BCVA returned to 20/25.

The course of self-limiting disease, coupled with characteristic ocular findings: a solitary choroidal granuloma with associated submacular SRD, prompted *Bartonella* serological evaluation following informed consent. An Immunofluorescence assay demonstrated elevated *Bartonella henselae* IgG titers (1:256; reference < 1:64).

A final diagnosis was established as CSD based on characteristic ocular findings, self-resolving disease course and serological evidence of *Bartonella henselae* infection.

The 11-week follow-up investigation revealed that full structural recovery confirmed by SD-OCT, including reconstitution of ellipsoid zone, outer photoreceptor segments, and interdigitation zone (Fig. 2F and L), and BCVA returned to 20/20.

## Discussion

We report a unique case of CSD characterized by *Bartonella henselae* infection without a history of feline exposure, presenting with isolated ocular manifestations. The patient developed visual impairment secondary to an isolated choroidal granuloma with SRD, notably lacking systemic symptoms. Remarkably, complete spontaneous resolution of the lesion and restoration of visual acuity were observed without targeted antimicrobial intervention for *Bartonella* infection.

The diagnosis of this case poses significant challenges due to clinical diversity. The differential diagnosis of choroidal mass encompasses systemic granulomatous disorders (e.g., sarcoidosis, tuberculosis, syphilis) [13–15], parasitic infections (ocular toxocariasis) [16], and localized inflammatory processes (focal scleral nodule) [17].

In the current case, imaging revealed an isolated choroidal nodular lesion with associated SRD, notably lacking hard exudates—a finding consistent with previous reports of macular-involved exudative detachments in CSD [18]. While multimodal imaging characterization of CSD-associated choroidal nodules remains infrequently documented, Sarra Gattoussi et al. [11] characterized choroidal granulomas in *Bartonella* infection as solitary or multifocal, unilateral/bilateral yellowish subretinal deposits. Angiographic analysis revealed late-phase hyperfluorescence on FA and hypofluorescent patterns on ICGA, with enhanced-depth SD-OCT demonstrating choroidal hyporeflective nodular lesions inducing retinal elevation. These imaging features were consistent with the findings in our case.

Notably, our patient’s presentation lacked two hallmark features of typical CSD neuroretinitis - macular exudative stars and optic disc edema - while simultaneously testing negative for epidemiological risk factors (feline exposure), and there were no systemic symptoms such as fever and lymphadenopathy. These atypical characteristics initially reduced clinical suspicion for *Bartonella* infection. The solitary choroidal mass case reported by Fariba Ghassemi et al. [19], also demonstrated spontaneous resolution without macular star formation, similar to our case.

Of note, the patient demonstrated increased subretinal fluid shortly after systemic steroid administration, with subsequent improvement following cessation of anti-inflammatory treatments. Although this clinical course may be consistent with the natural progression of the infectious process, it also serves as an important clinical reminder that systemic steroid monotherapy should be used with extreme caution in patients with suspected infectious chorioretinitis. Unnecessary steroid use without appropriate antimicrobial coverage may carry potential risks, and a conservative, watchful-waiting approach may be considered in clinically stable patients, given the self-limiting nature of this condition.

This report has several limitations. First, we did not perform Bartonella IgM testing, which limits definitive confirmation of acute infection. Second, the suboptimal quality of FA and ICGA images, likely due to older equipment, did not significantly impact the overall diagnostic impression. Despite these limitations, the combination of clinical, multimodal imaging, and serological findings remains highly consistent with a diagnosis of ocular CSD.

CSD, alternatively termed cat-scratch fever or benign lymphatic histiocytosis, represents an infectious pathology caused by *Bartonella henselae*. While primary transmission typically occurs through feline scratches, bites, or close contact with cats, alternative vectors exist. Intriguingly, ocular manifestations of CSD occasionally present in patients lacking documented feline exposure or arthropod bites [20]. Despite the absence of reported feline exposure in our case. At first presentation, the fundoscopic features were not pathognomonic for any specific condition. However, as the disease followed a self-limiting clinical course, this characteristic evolution led us to revisit the differential diagnosis and strongly suspect ocular CSD. Subsequent serological testing confirmed a markedly elevated IgG titer against *Bartonella henselae*, consistent with the diagnosis of ocular CSD.

Toshihiko Matsuo et al. [20] demonstrated CSD’s potential to manifest as submacular exudation with SRD. This presentation in our case is similar to the second case in Matsuo et al.‘s series, where diagnosis was serologically confirmed through elevated B. henselae titers despite lacking classical epidemiological markers (febrile episodes, lymphadenopathy, or feline contact). A distinctive feature of our case lies in the spontaneous resolution of lesions without targeted antimicrobial intervention for *Bartonella* infection, highlighting the spectrum of disease progression even in serologically confirmed infections. This clinical paradox is exemplified in Kawasaki et al.‘s seminal work [21], which emphasizes that *Bartonella* titres should be obtained whenever there is a clinical index of suspicion, regardless of cat exposure.

## Conclusion

This case highlights key features of atypical ocular CSD: (1) Self-Limiting isolated choroidal granuloma with SRD. (2) Multimodal imaging and *Bartonella* serology are essential when classic features (feline exposure, macular star) are absent. Observational management suffices for mild, self-resolving cases, as the condition may resolve spontaneously without targeted antimicrobial therapy, even without anti-inflammatory therapy. Early multimodal imaging and serologic testing reduce diagnostic delays. This report expands the ocular CSD phenotype, demonstrating that neither systemic symptoms nor classic ocular manifestations are prerequisites for diagnosis.

## Data Availability

The original contributions presented in the study are included in the article, further inquiries can be directed to the corresponding author.

## Abbreviations

BCVA: Best corrected visual acuity
CSD: Cat scratch disease
EST: Erythrocyte sedimentation rate
FA: Fluorescein angiography
FP: Fundus photography
ICGA: Indocyanine green angiography
SD-OCT: Spectral-domain optical coherence tomography
SRD: Serous retinal detachment
TB-IGRA: Tuberculosis interferon-gamma release assay
TOX: Toxoplasma gondii
